# Association of Age, Systolic Blood Pressure, and Heart Rate with Adult Morbidity and Mortality after Urgent Care Visits

**DOI:** 10.5811/westjem.2016.6.30353

**Published:** 2016-08-08

**Authors:** James Hart, Michael Woodruff, Elizabeth Joy, Joseph Dalto, Gregory Snow, Rajendu Srivastava, Brad Isaacson, Todd Allen

**Affiliations:** * Intermountain Healthcare, Intermountain Instacare, Salt Lake City, Utah; † Intermountain Healthcare, Intermountain Medical Center, Department of Emergency Medicine, Salt Lake City, Utah; ‡ Intermountain Healthcare, Quality and Patient Safety, Salt Lake City, Utah; § Intermountain Healthcare, Community Benefit, Salt Lake City, Utah; ¶ Intermountain Healthcare, Office of Research, Salt Lake City, Utah; || Intermountain Healthcare, Institute for Healthcare Leadership, Salt Lake City, Utah; # Intermountain Healthcare, Intermountain Medical Center, Department of Medicine, Salt Lake City, Utah; ** University of Utah School of Medicine, Department of Pediatrics, Salt Lake City, Utah

## Abstract

**Introduction:**

Little data exists to help urgent care (UC) clinicians predict morbidity and mortality risk. Age, systolic blood pressure (SBP), and heart rate (HR) are easily obtainable and have been used in other settings to predict short-term risk of deterioration. We hypothesized that there is a relationship between advancing age, SBP, HR, and short-term health outcomes in the UC setting.

**Methods:**

We collected retrospective data from 28 UC clinics and 22 hospitals in the Intermountain Healthcare system between years 2008–2013. Adult patients (≥18 years) were included if they had a unique UC visit and HR or SBP data. Three endpoints following UC visit were assessed: emergency department (ED) visit within three days, hospitalization within three days, and death within seven days. We analyzed associations between age, SBP, HR and endpoints using local regression with a binomial likelihood. Five age groups were chosen from previously published national surveys. Vital sign (VS) distributions were determined for each age group, and the central tendency was compared against previously published norms (90–120mmHg for SBP and 60–100bpm for HR.)

**Results:**

A total of 1,720,207 encounters (714,339 unique patients) met the inclusion criteria; 51,446 encounters (2.99%) had ED visit within three days; 12,397 (0.72%) experienced hospitalization within three days; 302 (0.02%) died within seven days of UC visit. Heart rate and SBP combined with advanced age predicted the probability of ED visit (p<0.0001) and hospitalization (p<0.0001) following UC visit. Significant associations between advancing age and death (p<0.0001), and VS and death (p<0.0001) were observed. Odds ratios of risk were highest for elderly patients with lower SBP or higher HR. Observed distributions of SBP were higher than published normal ranges for all age groups.

**Conclusion:**

Among adults seeking care in the UC, associations between HR and SBP and likelihood of ED visits and hospitalization were more pronounced with advancing age. Death following UC visit had a more limited association with advancing age or the VS evaluated. Rapidly increasing risk below SBP of 100–110 mmHg in older patients suggests that accepted normal ranges for SBP may need to be redefined for patients treated in the UC clinic.

## INTRODUCTION

Over the past several decades, urgent care (UC) clinics have become an increasingly popular venue among patients seeking unscheduled ambulatory care in the United States.^1^–^3^ The 2014 Urgent Care Association of America survey found that UC clinics manage nearly 78 million patient encounters per year within approximately 6,100 UC clinics (excluding retail clinics).^1^–^3^ Intermountain Healthcare, a vertically integrated healthcare delivery system has likewise observed a disproportionately larger increase in UC patient encounters between 2004 and 2013 of 91%, compared to a 14% increase in emergency department (ED) visits, and a 12% increase in primary care visits during that same time period. Given the increasing burden of chronic disease management in primary care,^4^–^6^ and consumer preference for convenience,^7^ this trend is likely to continue. However, despite the increase in UC visits, studies describing short-term clinical outcomes following evaluation and management in the UC remain limited.

A number of patients with severe illness have been observed to inappropriately present to UC, rather than the ED. As this phenomenon is likely to continue, timely and accurate identification of patients at risk for serious illness or adverse outcomes is critically important to ensure patient safety in the UC setting. With every visit, UC clinicians must make decisions to either discharge patients home, or to transfer them to a higher level of care such as the ED or hospital. Though many variables exist in patient triage, providers in the UC setting have traditionally relied upon abnormal vital signs (VS), particularly heart rate (HR) and systolic blood pressure (SBP), to assist in identifying patients with potentially acute, life-threatening illnesses. Normal ranges of vital signs including SBP and HR have been defined for both children and adults.^8^–^11^ While there is some variation among definitions, particularly the upper limit of normal vital signs, 90–120mmHg for SBP and 60–100bpm for HR are commonly cited.^12^–^15^ However, these ranges are based on a state of wellness and lack contextual relevance regarding age and other important variables (i.e. setting of care, chief complaint, disease burden, etc.), and thus limit their utility in clinical decision making.

While some studies take into account specific disease states and venues of care to help providers estimate risk of clinical decline based on vital signs, there may be limited applicability to the UC setting. First, prominent ambulatory guidelines regarding vital signs (e.g. SBP and hypertension) are designed to focus on longer-term outcomes such as myocardial infarction or stroke.^16^–^17^ Furthermore, short-term outcomes such as proximal death or intensive care unit (ICU) admission may be too limiting for measuring an appropriate margin of safety in the UC setting.^18^–^20^ Additionally, critical care models using vital signs (HR and SBP) and other relevant data to forecast short-term health outcomes may be challenging to apply to a heterogeneous patient population of the UC (as similarly described in the ED literature).^21^–^22^

Despite these limitations, advancing age has been defined as an independent risk factor for poor short-term health outcomes among those with sepsis, influenza, and pneumonia.^23^–^28^ Likewise, the relationship between SBP, age, and mortality has been examined in a recent trauma study.^29^ As such, we sought to further investigate the relationship between advancing age, vital signs (HR and SBP), and short-term health outcomes in the unscheduled ambulatory domain.

The objectives of our study were to (1) describe the age-specific distributions of HR and SBP observed in adults at the UC clinic, (2) define the short-term morbidity and mortality after a visit to an UC clinic, and (3) examine the association between age-specific vital signs and subsequent hospital utilization and/or mortality.

## METHODS

### Study Design

We performed a cross-sectional retrospective study by including adult patients (> 18 years old) cared for in 28 Intermountain Healthcare UC clinics and 22 hospitals between January 2008 and December 2013. The study was approved by the institutional review board.

### Data Source, Quality and Outcomes

Patient electronic health information is stored in Intermountain Healthcare’s enterprise data warehouse (EDW), a repository with over six million patient records.^39^ Each patient received a unique enterprise-wide identification that is used for every inpatient and outpatient encounter. This identifier was used to confirm an UC visit as defined by a billed encounter through our financial accounting system and was subsequently linked to the EDW to obtain subsequent resource utilization and clinical outcomes data. We included only UC encounters with a HR or SBP recorded in the electronic health record (EHR). Duplicate records of UC encounters were collapsed into a single visit.

Patients initially seen at an Intermountain Healthcare UC setting were followed for subsequent visits in any of the 22 EDs or hospitals during the study period. We defined outcomes of interest as an ED visit within three days, 30–33 hospitalization within three days,^30^–^33^ and death within seven days.^30^–^34^ Mortality was recorded in the EHR and further validated using data from the State Office of Vital Records.

The EDW was queried for demographic, vital sign, and clinical outcomes data. Systolic blood pressure was obtained by automated monitors (Dinamap or Phillips) or taken manually. Likewise, HR values were obtained by pulse oximetry, automated blood pressure/heart rate devices, or taken manually. All VS data were entered into discrete data fields in the EHR; non-discrete data were excluded in the data analysis. Furthermore, when more than one set of VS was recorded in a codified field within the EHR on the same day, we excluded the encounter due to uncertainty of the clinical setting where the vital sign was obtained.

We excluded cases if HR and SBP values were outside physiologic ranges (e.g. HR <0 or >300, SBP <0 or >400), or where there were obvious data quality issues such as diastolic blood pressure exceeding SBP. Review of extreme VS values suggested poor data quality and small sample sizes; therefore, the dataset was further limited to HR of ≥30bpm and ≤180 bpm and SBP of ≥60mmHg and ≤240mmHg. Two authors manually reviewed data in two phases. In the first phase > 200 charts were randomly identified and the designated author reviewers performed iterative cycles of data validation to ensure that the VS were correctly attributed to the UC visit, and the outcomes (ED visits, hospitalizations, and deaths) were accurate. Secondly, a final data validation was manually performed on all deaths (302 encounters).

To assess the impact on advancing age on health outcomes following an UC visit, we chose five age groups based on the Centers for Disease Control and Prevention’s National Hospital Ambulatory Medical Care Survey^35^ (Group 1: 18–24, Group 2: 25–44, Group 3: 45–64, Group 4: 65–74, Group 5: 75+).

### Statistical Analysis

We fit a local regression with binomial likelihood to explore the relationship between advancing age, vital signs (HR and SBP), and short-term health outcomes in the unscheduled ambulatory domain.^36^ Graphics were generated to display the age-adjusted risk for each outcome measure based on HR and SBP. Additionally, data were presented as an odds ratio table for hospitalization. To further understand and categorize age-specific distributions on HR and SBP prevalence in the UC setting, we computed the central tendency for the five age groups. All data were analyzed using R (version 3.1.1, Vienna, Austria).

## RESULTS

**Figure 1:**
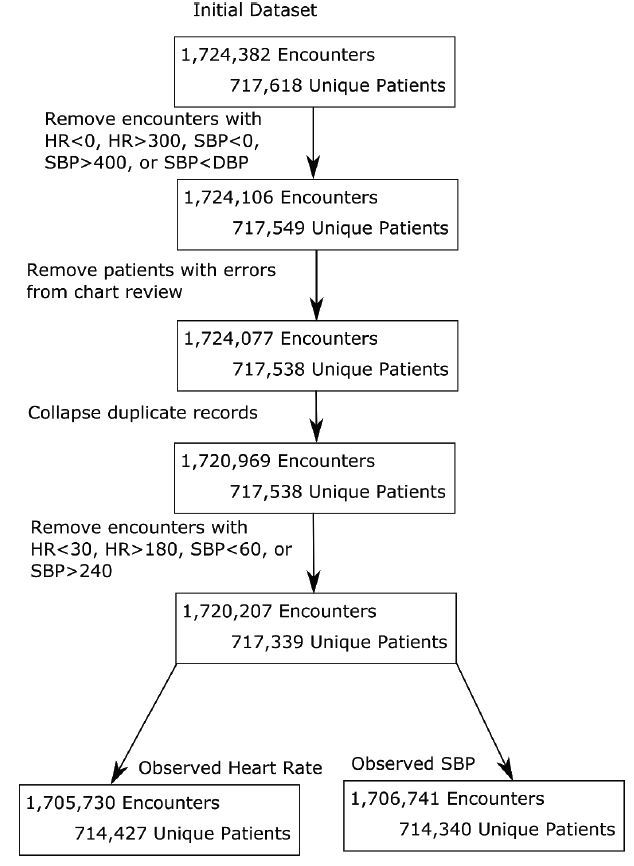
Workflow of patient inclusion/exclusion. *HR,* heart rate; *SBP*, systolic blood pressure; *DBP*, diastolic blood pressure.

We initially included a total of 1,724,382 patient encounters (717,618 patients) in the data analysis; after applying the exclusion criteria noted above, this resulted in 1,720,207 encounters (717,339 patients) for final analysis. Because some patient encounters had either only HR measures or SBP measures (the vast majority had both), we finally segmented the data into 1,705,730 encounters (714,427 patients) for HR and 1,706,741 encounters (714,340 patients) for SBP in order to allow for VS analysis (Figure 1).

A total of 51,446 UC encounters (2.99%) were followed by an ED visit within three days, 12,397 encounters (0.72%) resulted in hospitalization within three days, and 302 (0.02%) were associated with death within seven days. The average age was 42±18 years old. Females represented 59.72% of subjects (1,027,387 encounters, 394,822 patients), 40.28% were male (692,809 encounters, 322,510 patients), and 11 encounters (7 patients) did not have gender recorded. Females averaged 2.60 UC encounters per patient, and males averaged 2.15 UC encounters per patient during the study period.

**Figure 2:**
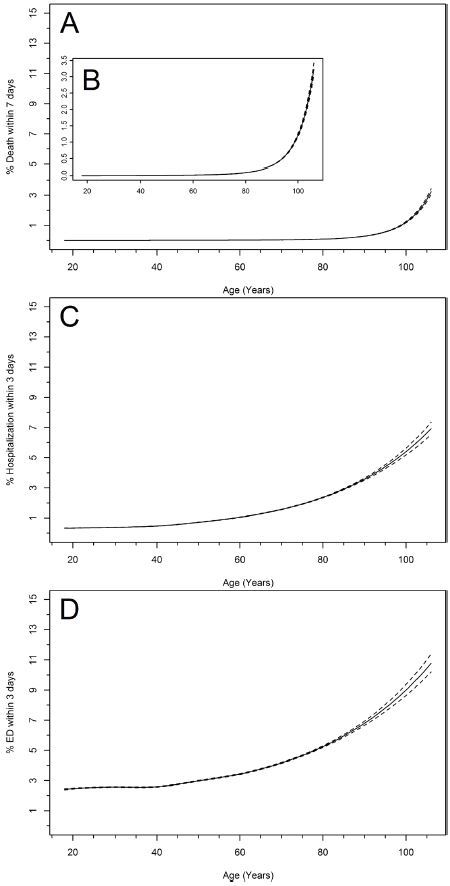
Age-specific risk curves for morbidity and mortality subsequent to an urgent care (UC) visit. Part A shows mortality within 7 days of an UC visit, magnified in part B. Part C shows percent hospitalization within 3 days and part D shows percent emergency department visits within 3 days of an UC visit. The solid line represents point estimates and the dotted lines are 95% confidence intervals.

**Figure 3:**
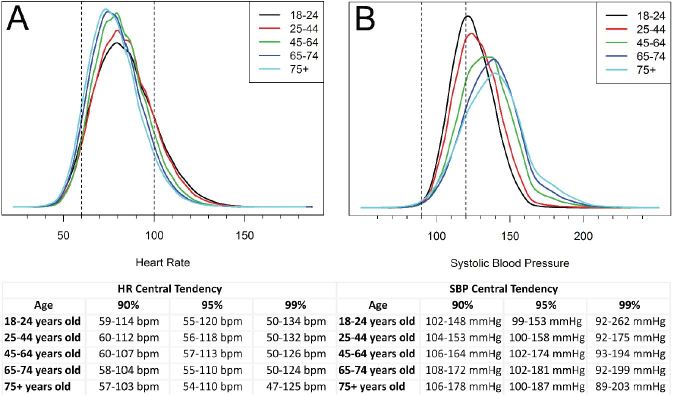
Distributions of observed values of heart rate (HR) (A) and systolic blood pressure (SBP) (B) for different age groups. Generally accepted “normal” ranges are indicated by vertical dashed lines.^12^–^15^ The tables below the graphs show the 90th, 95th and 99th percentiles by age group. Aggregate HR data fit within the “normal” range approximately 82% of the time for HR, but only about 31% for SBP. *bpm*, beats per minute; *mmHg*, millimeters of mercury

Risk for ED visit, hospitalization and death increased with age in a curvilinear pattern (Figure 2). When the 90th percentile was computed for observed HR values, Group 1 (18–24 years old) ranged between 59–114 bpm, Group 2 (25–44 years old) was 60–112 bpm, Group 3 (45–64 years old) was 60–107 bpm, Group 4 (65–74 years old) was 58–104 bpm, and Group 5 (75 + years old) was 57–103 bpm (Figure 3A). HR values that fit within published normal ranges occurred 82% of the time within our dataset, and increased to 86% when considering advanced age (Groups 4 and 5).

The 90th percentile for SBP ranged from 102–148 mmHg for Group 1, 104–153 mmHg for Group 2, 106–164 mmHg for Group 3, 108–172 mmHg for Group 4, and 106–178 mmHg for Group 5 (Figure 3B). Only 31% of SBP values in this dataset fit within published normal ranges for the age groups; this decreased to 18% when considering advanced age (Groups 4 and 5). Furthermore, a SBP less than 99 mmHg was a rare observation in all age groups (<95^th^ percentile), even more so, a SBP of 90mmHg (≤ 99^th^ percentile) suggesting that in the UC setting any SBP value < 100mmHg should be viewed cautiously.

**Figure 4:**
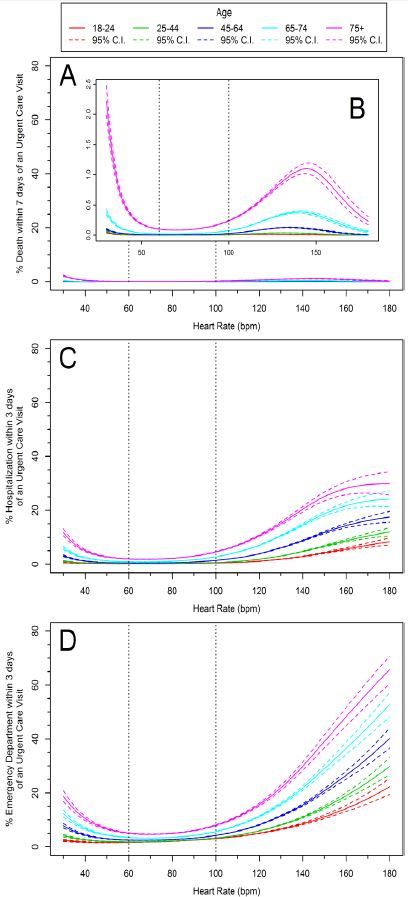
Age-specific heart rate (HR) risk curves for morbidity and mortality subsequent to an urgent care (UC) visit. Part A shows death within 7 days of an UC visit, magnified in part B. Part C shows percent hospitalization within 3 days of an UC visit, and Part D shows percent emergency department visits within 3 days of an UC visit. Published “normal” ranges are noted by vertical dashed lines.^12^–^15^ The solid lines represent point estimates and the dotted lines are 95% confidence intervals. *bpm,* beats per minute.

**Figure 5:**
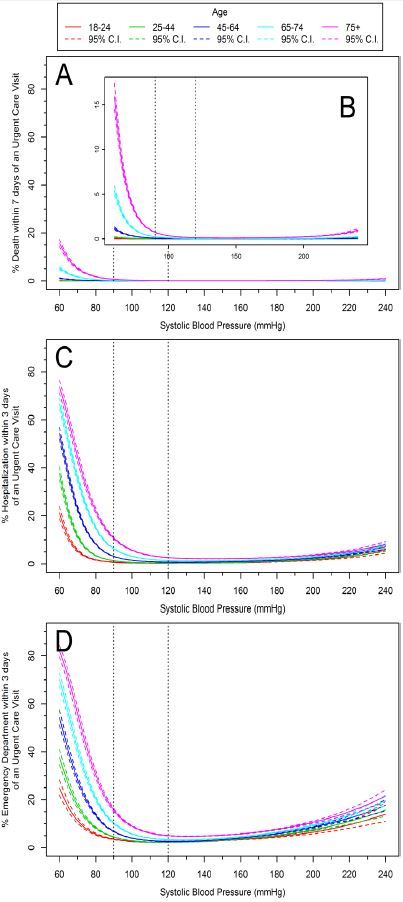
Age-specific systolic blood pressure (SBP) risk curves for mortality and morbidity subsequent to an urgent care (UC) visit. Part A shows percent death within 7 days of an UC visit, magnified in part B. Part C shows percent hospitalization within 3 days of an UC visit, and part D shows percent hospitalization within 3 days of an UC visit. Published “normal” ranges are noted by vertical dashed lines.^12^–^15^ The solid lines represent point estimates and the dotted lines are 95% confidence intervals. *mmHg,* millimeters of mercury.

Vital signs (HR and SBP) and advancing age demonstrated curvilinear associations with the likelihood of an ED visit and hospitalization (p<0.0001) (Figures 4 and 5). We observed significant associations with advancing age and death, or vital signs (HR or SBP) and death (p<0.0001). However, outcomes between age and HR with death, and age and SBP with death were not statistically significant (p=0.66 and p=0.07 respectively).

The influence of age on the relationship between vital signs (HR and SBP) and hospitalization is further illustrated in Tables 1 and 2. For example, in patients with a HR between 60–100 bpm, a nearly 10-fold greater likelihood of hospitalization was observed in patients 75 years of age or older compared to patients 18–24 years of age (OR 10.57, 95% CI [9.6–11.7]). We observed a similar relationship for SBP of 100–120 mmHg in patients 75 years of (OR = 11.83, 95% CI [10.3–13.6], Tables 1 and 2).

## DISCUSSION

To our knowledge, this is the largest published study of UC encounters and subsequent short-term outcomes. Predictor variables and the outcomes of interest were selected based on the potential for increased downstream healthcare utilization and/or clinical deterioration. This study found that approximately 3% of UC visits had a subsequent escalation of care or death, with the vast majority comprised of ED encounters. While this is consistent with a national survey of UC visits (78% response rate) describing 4% or less rate of transfer to the ED,^2^ the comparison is limited due to the fact that a significant number of our three-day ED visits were likely not intended transfers. Comparisons for our rate of hospitalization (0.72%) and death (0.02%) are unavailable due to lack of published data. Therefore, future research will be needed to establish more accurate baselines for outcomes following an UC visit.

Data demonstrated that the distribution of observed HR values varied little between different age groups, and was generally centered within the accepted normal range for adults. Older patients tended toward a narrower distribution with lower heart rates than younger patients. This is not surprising since HR tends to decrease with age.^38^ The relationship between age-stratified HR and short-term outcomes was also curvilinear, with a more pronounced effect above 90–100 bpm. This suggests that in the unscheduled ambulatory setting, heart rates of 90–100 may be indicators of risk, especially in older patients.

The distribution of observed SBP values in our population varied between age groups, with older patients tending toward a higher SBP. Interestingly, the distribution of SBP observed in all age groups were higher than published normal ranges (Figure 3B). However, the risk of short-term deterioration was not as profound for hypertensive patients as it was for patients with lower SBP. The relationship between lower SBP and short-term deterioration was most pronounced among older patients, in which the risk began to increase more rapidly below a SBP of approximately 100–110mmHg—well above “low normal”.^37^ These findings suggest that for older patients seeking unscheduled care in the UC clinic, the safe lower limit of SBP may need to be redefined.

Taken together, these findings suggest that rather than relying upon textbook “normal” ranges for adult vital signs to estimate clinical risk, UC providers may find venue-specific data relating VS ranges to short-term outcomes more useful in medical decision-making. For example, when evaluating an elderly patient with an acute complaint, the provider may not view a SBP of 105 as “nice and low” but instead may understand that the patient may be at an inflection point on the risk curve, and could evaluate more closely other indicators of impending deterioration.

The idea that VS measurement and interpretation require context is one that has gained some attention, but this is the first time that the “context” of an UC visit has been related to vital signs and short-term health and utilization outcomes. Additional work is needed to validate these findings and to seek to provide a better understanding of the nature and distribution of vital signs in other healthcare venues.

## LIMITATIONS

There are several limiting factors worth noting in this study. While this is a very large dataset, and Intermountain Healthcare treats approximately 50% of the state’s population, our findings are based on only one healthcare system and may not be generalizable to other systems. Some of our outcome data may be considered incomplete because our visit outcomes of interest could not be collected for patients who received care at other facilities and death records were not obtained from outside of our state. Additionally, at the time of this study, our EHR/EDW did not capture whether a visit to the ED or hospital after an UC visit was intended (i.e. the result of a transfer) or unintended. A proportion of these patients may have been referred to the ED or directly admitted to the hospital, but this cannot be determined from this dataset. This categorization could be useful in describing unanticipated short-term health outcomes and will be important in future reviews. Additionally, we excluded a small number of encounters with extreme vital signs from our analysis. While patients do rarely present to the UC clinic with HR <30 bpm or >180 bpm, and/or with SBP of <60mmHg or >240mmHg, the risk associated with these vital signs should be immediately apparent to the provider and guide appropriate treatment.

The scope of this paper was limited to vital signs and age. Because of this, we did not examine other possible predictor variables, such as disease burden, medications, chief complaint, treatment provided, prior healthcare utilization, payer type, leaving against medical advice/elopement, time of day, seasonality, or provider type. Further research will be needed to understand the interaction between risk factors, specific disease processes and clinical deterioration in the UC setting.

## CONCLUSION

In this large cohort of UC encounters, there were associations between advancing age, vital signs (HR and SBP), and likelihood of ED visits and hospitalization following an UC visit. An association was also observed between advancing age or vital signs (HR or SBP) and death. While SBP values between 90–100 mmHg are commonly referenced as the lower limit of normal in healthy adults, SBP values <100 mmHg were uncommon in this cohort, and were significantly related to adverse short-term health outcomes. Published normal ranges for SBP (90–120 mmHg) may need to be redefined for adult patients seen in the UC setting.

## Figures and Tables

**Table 1:** Odds ratio table of heart rate ranges and hospitalization within 3 days subsequent to an urgent care visit, subsegmented by age. Note, the top number in each row denotes the odds ratio, the second is the 95% confidence internal, and the last is the sample size within each grouping.

Heart Rate (bpm)						

			<60 bpm	60–100	101–129	130+
			
Age (years)	18–24	OR	0.77	1.00	3.31	17.55
		CI (95%)	0.5–1.1	N/A	2.9–3.8	14.1–21.8
		n	16,247	35,331	45,273	2,745

	25–44	OR	1.19	1.35	4.67	16.52
		CI (95%)	0.9–1.5	1.2–1.5	4.2–5.2	13.9–19.7
		n	34,512	619,284	105,487	5,008

	45–64	OR	2.22	2.89	11.54	51.40
		CI (95%)	1.8–2.8	2.6–3.2	10.4–12.9	42.1–62.7
		n	19,134	362,062	41,619	1,344

	65–74	OR	4.24	5.48	20.90	121.51
		CI (95%)	3.3–5.5	4.9–6.1	18.2–23.9	92.3–159.9
		n	7,220	100,256	8,637	350

	75+	OR	9.82	10.57	33.41	104.01
		CI (95%)	8.2–11.8	9.6–11.7	29.3–38.1	78.2–138.3
		n	7,648	86,740	6,479	354

*bpm*, beats per minute; *OR*, odds ratio; *CI*, confidence interval; *n*, sample size; *N/A,* not applicable

**Table 2:** Odds ratio table of systolic blood pressure ranges and hospitalization within 3 days subsequent to an urgent care visit, subsegmented by age. Note, the top number in each row denotes the odds ratio, the second is the 95% confidence internal, and the last is the sample size within each grouping.

Systolic Blood Pressure (mmHg)								

			<80 mmHg	81–89	90–99	100–120	121–180	180+
			
Age (years)	18–24	OR	9.76	6.39	1.59	1.00	1.15	1.89
		CI (95%)	3.5–27.1	3.4–11.9	1.1–2.3	N/A	1.0–1.3	0.3–14.1
		N	142	591	7,432	116,227	174,311	179

	25–44	OR	31.50	6.15	2.20	1.32	1.41	5.60
		CI (95%)	20.1–49.4	4.0–9.5	1.7–2.8	1.2–1.5	1.3–1.6	4.0–7.9
		N	269	1,282	15,268	246,078	500,120	2,383

	45–64	OR	82.30	27.96	8.55	2.82	2.62	7.35
		CI (95%)	57.6–117.6	20.9–37.3	7.0–10.4	2.5–3.2	2.3–2.9	6.0–9.0
		N	219	783	5,780	91,912	319,683	6,511

	65–74	OR	116.78	55.90	19.18	6.21	4.30	10.02
		CI (95%)	73.1–186.4	38.7–80.7	14.8–24.8	5.3–7.2	3.8–4.9	7.9–12.8
		N	101	267	1,448	19,389	92,220	3,048

	75+	OR	105.63	50.69	24.95	11.83	7.91	11.33
		CI (95%)	71.4–156.3	36.5–70.4	20.1–31.0	10.3–13.6	7.0–8.9	9.2–13.9
		N	155	367	1,755	17,065	77,638	4,118

*mmHg*, millimeters of mercury; *OR*, odds ratio; *CI*, confidence interval; *n*, sample size; *N/A,* not applicable
